# Species-Independent Down-Regulation of Leaf Photosynthesis and Respiration in Response to Shading: Evidence from Six Temperate Tree Species

**DOI:** 10.1371/journal.pone.0091798

**Published:** 2014-04-11

**Authors:** Anping Chen, Jeremy W. Lichstein, Jeanne L. D. Osnas, Stephen W. Pacala

**Affiliations:** Department of Ecology and Evolutionary Biology, Princeton University, Princeton, New Jersey, United States of America; Institute of Botany, Chinese Academy of Sciences, China

## Abstract

The ability to down-regulate leaf maximum net photosynthetic capacity (Amax) and dark respiration rate (Rdark) in response to shading is thought to be an important adaptation of trees to the wide range of light environments that they are exposed to across space and time. A simple, general rule that accurately described this down-regulation would improve carbon cycle models and enhance our understanding of how forest successional diversity is maintained. In this paper, we investigated the light response of Amax and Rdark for saplings of six temperate forest tree species in New Jersey, USA, and formulated a simple model of down-regulation that could be incorporated into carbon cycle models. We found that full-sun values of Amax and Rdark differed significantly among species, but the rate of down-regulation (proportional decrease in Amax or Rdark relative to the full-sun value) in response to shade was not significantly species- or taxon-specific. Shade leaves of sun-grown plants appear to follow the same pattern of down-regulation in response to shade as leaves of shade-grown plants. Given the light level above a leaf and one species-specific number (either the full-sun Amax or full-sun Rdark), we provide a formula that can accurately predict the leaf's Amax and Rdark. We further show that most of the down regulation of per unit area Rdark and Amax is caused by reductions in leaf mass per unit area (LMA): as light decreases, leaves get thinner, while per unit mass Amax and Rdark remain approximately constant.

## Introduction

Plant photosynthesis and respiration are critical components of carbon cycle models from individual to global scales [1]–[4]. Maximum (high light) rates of photosynthesis (Amax) and leaf dark respiration (Rdark) are two important determinants of net primary productivity (NPP) [5]. Mechanistic models of the carbon cycle either assume these quantities as parameters or predict them from submodels. An important challenge for modelers is that Amax and Rdark vary widely within and between individuals and species in response to environmental factors such as light level [6], [7].

Light is a critical resource controlling the carbon budget of plant photosynthesis and respiration [8], [9], and many studies have examined leaf Amax and Rdark in contrasting light environments (e.g., Sims and Pearcy [10]). Reduced Amax and Rdark in response to shade has been observed in many species [6], [10], [11] and constitutes an important way for plants to cope with the extreme heterogeneity in light levels they may experience. However, most empirical studies focus on seedlings in a few light levels in a greenhouse [6], [10]–[12]. Thus, there is limited information on sapling Amax and Rdark across the full range of light conditions experienced in natural and semi-natural forests, which limits our capacity to test and improve canopy integration schemes used in carbon cycle models [13], [14]. Limited information on how sapling Amax and Rdark vary within and between individuals and species is particularly problematic for next-generation global vegetation models that represent individual-level height-structured competition [3], [15], because sapling understory performance exerts a strong impact on forest dynamics [16].

In this paper, we make early steps towards a simple model of down-regulation on Amax and Rdark within and between species and within individuals, formulated at a level that would be useful in carbon cycle models. Here we define “down-regulation” as a decrease in leaf-level Amax and/or Rdark rates with decreasing light availability, regardless of the physiological or morphological mechanisms involved. We focus on differences between sun- and shade-grown leaves, rather than acclimation by individual leaves exposed to different light levels. Down-regulation may occur due to molecular or cell-level biochemical processes, as well as leaf-level morphological adjustments such as reductions in leaf mass per unit area [14, and references therein]. We base the model on a novel dataset and statistical framework. We measured light saturated leaf Amax and Rdark for saplings of six temperate tree species differing in shade tolerance and life history. For each species, we measured Amax and Rdark across the full range of light conditions experienced by individual saplings and leaves in nature, including high- and low-light leaves from trees acclimated to high-light, and leaves from trees acclimated to shade. For five of the six species, we also measured light availability for individual saplings. We used the data to develop a simple statistical model to explain within and between species patterns of Amax and Rdark down-regulation in response to shade. Our model provides simple rules that could be incorporated into models of forest carbon dynamics if subsequent research shows that they apply beyond our sample of six species.

## Methods

### Species

We measured Amax and Rdark for leaves of six tree species from temperate forest in New Jersey, United States of America. These include two conifer species: eastern white pine (*Pinus strobus*) and eastern hemlock (*Tsuga canadensis*); and four angiosperm species: gray birch (*Betula populifolia*), white ash (*Fraxinus americana*), sugar maple (*Acer saccharum*), and American beech (*Fagus grandfolia*). Gray birch, white ash and white pine are shade intolerant; and eastern hemlock, sugar maple and American beech are shade tolerant [17], [18].

### Field sites

Most individuals were sampled at Princeton University's Stony Ford Field Station (40°21.24′N, 74°43.3′W) and Stokes State Forest (41°11.25′N, 74°47.9′W). In addition, Rdark was measured on four eastern hemlocks at Hacklebarney State Park (40°45.9′N, 74°43.0′W). To our knowledge, these sampling locations were not affected by anthropogenic irrigation or fertilization in the past. Sampling size of each species and measurement can be found in Table S1. Permission to work at the New Jersey state properties was granted by the New Jersey Department of Environmental Protection, Division of Parks and Forestry.

### Measuring Amax and Rdark

Amax and Rdark were measured on leaves on detached branches (see below) with a LI-6400 (Li-Cor Instruments) gas-exchange system. Given that most of the saplings we measured were taller than 5 m and spaced more than 10 m apart from each other, *in situ* gas exchange measurements for a large sample of individuals would have been impractical. Gas-exchange measurements on detached branches have been commonly reported across many tree species, including the birches, maples, beeches, ashes, pines and hemlocks studied here [19]–[23]. Numerous studies have reported no significant difference between detached and attached leaves for Amax (half hour) and Rdark (several hours) if detached branches are provided with sufficient water supply [24], [25]. Mitchell et al. [21] found that Rdark was stable for up to 6 hours following detachment. All of our Rdark measurements were taken within 6 hours of detachment, except for some gray birch measurements taken 6–7.5 hours after detachment (these were not significantly different from gray birch Rdark measurements taken within 6 hours of detachment; P = 0.4). In light of the above, we assume that our Amax and Rdark measurements for leaves from detached branches (details below) are representative of leaves from attached branches, but we acknowledge that this assumption is difficult to verify in the absence of direct comparisons between detached and attached branches at our study site (see also Discussion).

Amax measurements were light saturated and under ambient CO_2_ concentration. For each species, we sampled three categories of mature leaves from saplings about 2.5 to 10 m tall during mid summer: (a) upper canopy “sun” leaves from healthy saplings grown in full sun; (b) lower canopy “shade” leaves from the same saplings as in (a); and (c) leaves from suppressed understory saplings with very low direct and indirect light irradiance. By “suppressed”, we mean that the saplings were shaded and (except for American beech, which reproduces clonally) appeared with few leaves remaining. Because we could not find such saplings of American beech at our sites, we report values for heavily shaded individuals with a full complement of leaves. These three categories of leaves (“sun”, “shade” and “suppressed”) were assigned based on careful visual assessment with the aim of sampling a broad range of light environments and physiological conditions (from healthy to nearly dead). However, these categories were not intended as a quantitative index of light availability, which was measured for each individual (see the method section “Light availability”). For each of the three leaf categories, we sampled four to six saplings. Hemlock wooly adelgids (*Adelges tsugae*), a homopteran pest that infests eastern hemlock leaves, were gently removed so that their respiration would not affect the gas exchange measurements.

Amax was measured on sunny days between 10:00 a.m. and 1:00 p.m. This logistical constraint resulted in a smaller sample size for Amax than for Rdark. The LI-6400 system equipped with a clear plastic conifer chamber head was placed in full sun on a tripod near the sapling to be sampled. A one to two meter long branch containing the leaves to be measured was harvested and immediately transported to the LI-6400 system. The bottom of the branch was re-cut under water, removing about 50 cm of stem, and then kept in a tub filled with water throughout the measurement. One broad leaf or one bunch of conifer needles was placed in the chamber and directed toward the light. Note that natural sunlight was used in measuring Amax to best represent the quality of actual light the measured saplings are acclimated to. Many previous studies [26]–[28] have indicated a saturation of photosynthesis when photosynthetically active radiation (PAR) reaches >800–1200 µmol photons/m^2^/second. In this study, the measured PAR varied within 1318–2179 µmol photons/m^2^/second, with a mean value of 1825 µmol photons/m^2^/second, suggesting a light-saturated environment for all the photosynthetic gas exchange. Therefore the variation in PAR here should have little impact on the non-light-limited Amax. The reference CO_2_ flux control (i.e., the incoming CO_2_ stream that leaves are exposed to) was set at 400 µmol mol^−1^, the humidity in the chamber was set near ambient at 20 mmol mol^−1^. The chamber block temperature instead of leaf temperature was controlled at 25°C to ensure a fast stabilization of the leaf gas exchange. Amax and stomatal conductance (g_s_) were recorded when the gas exchange rate stabilized, which happened after approximately two to five minutes. To guard against anomalous data from damaged branches, our analysis only includes measurements from leaves whose gas exchange rates remained stable for an additional five minutes following the initial two-to-five minute stabilization period.

While it is a common practice to control the block temperature in gas exchange measurement [29], [30], it is worthwhile to note that such an approach could introduce variation to the measured leaf and air temperatures. In this study, the measured leaf temperature varied around 31.4°C (29.4–32.6°C) and the measured air temperature varied around 27.2°C (26.7–28.5°C). The variation in measured leaf temperature could cause potential bias in inter-specific comparisons of Amax. Previous studies have shown that the optimal temperature for leaf carbon assimilation is closely associated with the environmental temperature the plant is grown in (see the review by Berry and Björkman [31]), and for many temperate plant species acclimated to a photosynthetically active temperature around 25°C, the optimal temperature is usually above 30°C, mostly within 30–35°C [32]. Hence, the measured leaf temperature variation around 31.4°C (29.4–32.6°C) here should be close to their optimal carbon assimilation temperature, and the bias caused by the temperature variation should be limited.

While waiting for the Amax measurements to stabilize, roughly 10 to 20 broad leaves or conifer twigs with needles were removed from the same branch for subsequent Rdark measurements. These leaves were sealed in a plastic bag with a wet paper towel and stored in a cooler on ice. Before Rdark measurements, leaves were taken out of the cooler and put in a dark drawer or closet to warm to ambient temperature (∼21°C) for approximately 30 minutes. The chamber setup and methods for Rdark measurements were the same as those for Amax, except that Rdark was measured inside a building with little natural light. Respiration was measured no more than 6 hours after leaves were harvested, except for some gray birch leaves at remote sites that were measured 6–7.5 hours after harvest. Because Rdark was measured after less than a full night-length of darkness (a common procedure), the Rdark values we report may not be directly comparable to some values reported in the literature. However, because we used a consistent protocol for all leaves, the length of pre-measurement darkness should not affect the inter- and intraspecific comparisons that are the focus of our paper. Because we could not reliably measure gas exchange for eastern hemlock needles (due to their small size, ∼1–2 cm length), we measured Amax and Rdark for foliated twigs of this species, then removed the needles and repeated the measurement for twigs only. We subtracted the twig-only value from the twig-plus-needles value to estimate Amax and Rdark. It is common to remove the attached needles for twig respiration [33]. Note this is likely to induce a damage repair response, increasing the measured twig respiration rate and therefore reducing the derived needle respiration rate. However, given the much lower rate of twig respiration compared to needle respiration, this effect should be limited. After gas-exchange measurements, sampled leaves were scanned with a CanonScan LIDE 70 flatbed scanner, and leaf area (projected leaf area for white pine) was calculated using ImageJ 1.43 software (http://rsbweb.nih.gov/ij/). Leaves were then oven-dried at 60°C to constant mass and weighed. Amax and Rdark were recalculated per unit leaf area (Amax_area_ and Rdark_area_) and per unit leaf mass (Amax_mass_ and Rdark_mass_) using the acquired leaf area and mass data respectively.

### Light availability

A hemispherical photograph was taken in the field above the crown of each sapling of all species except for American beech. Photographs were recorded and processed using the WINSCANOPY system (Regent Instruments), which includes a Nikon Coolpix 4500 digital camera, a hemispherical lens, a self-leveling camera mount (which we attached to an extendable pole), and image analysis software. In this paper, we use the proportion of diffuse above-canopy PAR that is incident on a sapling's crown as an index of light availability. This index is highly correlated with integrated growing season PAR [34]. For each sapling, we obtained an estimate of this index from WINSCANOPY software calibrated against above- and below-canopy photon sensor measurements taken under overcast conditions (J.W. Lichstein, unpublished data).

### Empirical Models of Down Regulation

To test hypotheses about down-regulation and to develop a simple model of its action, we fit nested empirical models (for both mass- and area-based Amax and Rdark) to the data using maximum likelihood methods and evaluated them with likelihood ratio tests and the Akaike Information Criterion [35]. Suppose that:
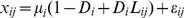
(1)where x_ij_ is the mass- or area-based Amax or Rdark of a leaf of individual-j of species-i; L_ij_ is the proportion of full sun directly above the leaf; μ_i_ is the mean Amax or Rdark of a species-i leaf in full sun (i.e. the maximum value); ε_ij_ is a normally distributed error; and D_i_ ranges between zero and one and measures the capacity of species-i to down regulate as light decreases. Species-i's full-sun rate of μ_i_ is reduced by a fraction D_i_, as L_ij_ is reduced from full sun (L_ij_ = 1) to complete shade (L_ij_ = 0). Our down-regulation index, D_i_, is a standardized slope of reaction norm [36] that predicts Amax or Rdark of a leaf given its light availability and the species-specific value μ_i_. We used maximum likelihood methods to fit nine models representing the nine possible combinations of: (1) a single value of μ shared by all species, (2) separate μ for angiosperm and conifer species, (3) species-specific μ, (a) a single value of D for all species, (b) separate D for angiosperm and conifer species, and (c) species-specific D. Note that all models with a single value of D (1a, 2a, and 3a) imply a single down-regulatory response for all species in a proportional sense; i.e., the expected ratio of x_ij_ to μ_i_ is (1−D+DL_ij_), which has no species-specific quantities. Likelihood ratio tests comparing two models were used to test if μ or D or both are taxon- or species-specific.

We calculated 95% confidence limits using the likelihood-profile method [35]. If the 95% confidence region for D from a model does not include zero, then we can reject the null hypothesis of no down regulation (D = 0) at P<0.05. We omitted the shade leaves of sun-grown saplings when fitting the models because we measured light levels immediately above the sun leaves of these individuals but not above the shade leaves. Thus, data on deeply shaded leaves in the model estimation came only from the tops of the deeply shaded saplings.

To evaluate how much of the down-regulation of per-unit-area values was caused solely by adjustment of leaf mass per area (LMA), we developed one other simple model:
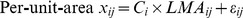
(2)where the species-specific constant C_i_ is Amax_mass_ or Rdark_mass_ for species-i, which is assumed to be a constant and independent of light level. The model will fit poorly if substantial down regulation per-unit mass occurs, and the model will fit well if down-regulation per-unit area is due solely to decreasing leaf thickness with decreasing light.

### Power analysis

Analysis of Equation 1 suggested that Model 3a (species-specific μ, but a single value of D shared across all species) was the best model (see Results). Failure to detect significant interspecific differences in D provides strong evidence against such differences only if statistical power to detect such differences is high. We conducted a power analysis to determine the probability of correctly identifying species differences in D, given the observed level of random error in the data (ε in Equation 1). The true underlying model in the power analysis was Model 3c (species-specific μ and species-specific D in Equation 1), and we quantified how the probability of correctly identifying Model 3c (as opposed to Model 3a) as the best model varied with (i) the level of interspecific difference in D, (ii) sample size, and (iii) the distribution of sampled light levels (either the actual distribution in our sample, or a uniform distribution ranging from zero to full sunlight). A detailed description of the power analysis is in the online Text S1.

## Results

### 1. Full-sun values of Amax and Rdark (μ_i_) differ significantly among species but the proportional rate of down regulation (D_i_) is not significantly species- or taxon-specific

**Figure 1:**
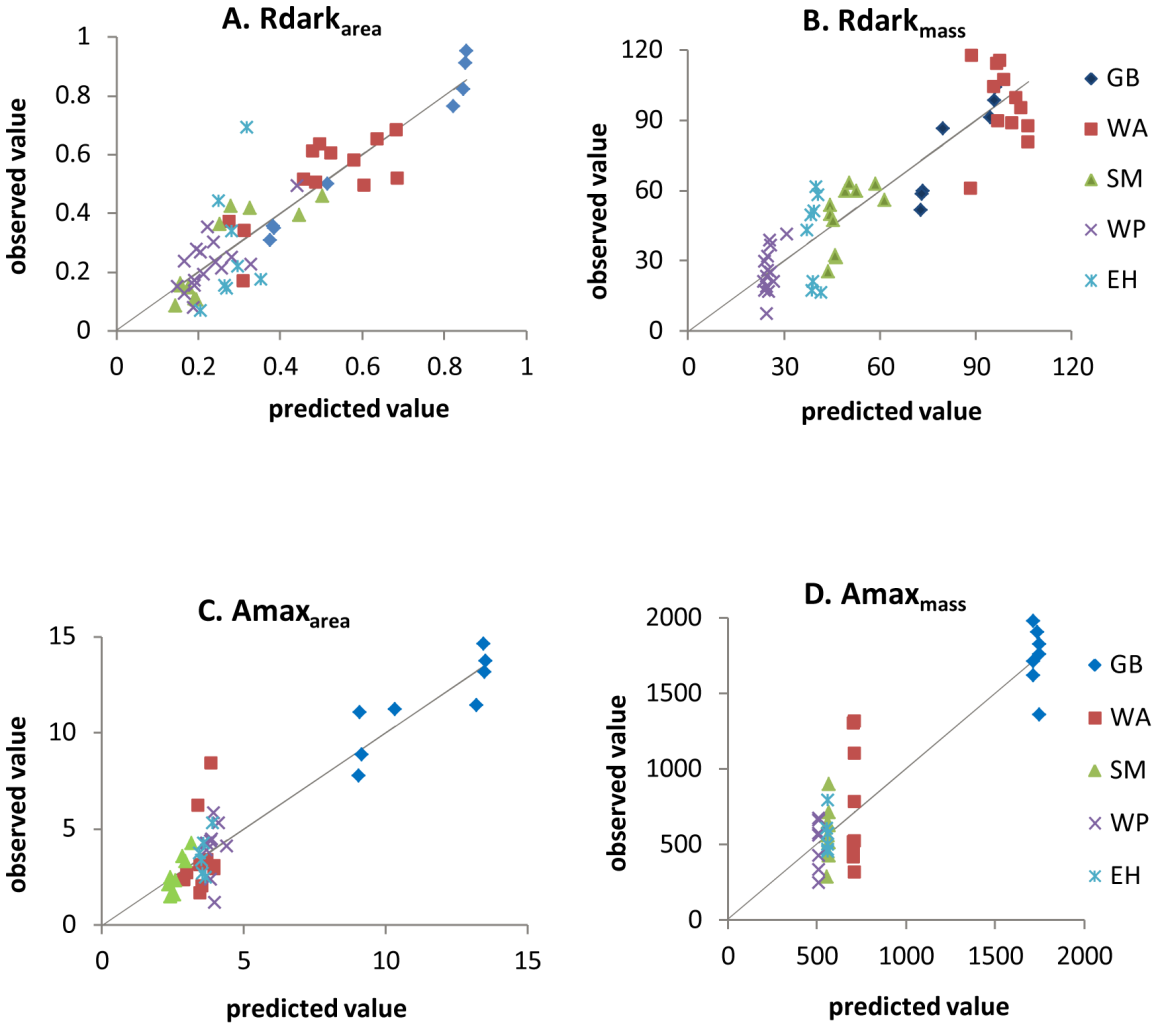
Measured vs. predicted values from Model 3a (universal D and species-specific μ). Dash lines are 1∶1. The model explains between-species variation in all four cases (area- and mass-based Amax and Rdark). The amount of within-species variation explained is greatest for Rdark_area_ (Figure 1a) and least for Amax_mass_ (Figure 1d). Units for per-area Amax and Rdark are µmol CO_2_ m^−2^ s^−1^, and units for per-mass Amax and Rdark are 10^−4^ µmol CO_2_ g^−1^ s^−1^. Species code: GB = gray birch, WA = white ash, SM = sugar maple, WP = white pine, EH = eastern hemlock.

Table 1 contains results for the maximum likelihood analysis of the nine models based on Equation (1). In most cases, models for per-unit-area data fit better than models of per-unit-mass data. The best overall model had species-specific μ and one value of D common to all species (Model 3a). In three of four cases (Amax_area_, Amax_mass_, and Rdark_area_), Model 3a had the lowest AIC, and its log likelihood was significantly higher than models with one value of μ shared by all species or with one value for angiosperms and another for conifers (p<0.05, likelihood ratio tests). In the fourth case (Rdark_mass_), the AIC of Model 3a is insignificantly higher than that of Model 1c (single value of μ and species-specific D). Also, Model 3a's log likelihood for Rdark_mass_ was insignificantly lower (p>0.05) than models with species- or taxon-specific D's and species-specific μ's. In summary, there is strong evidence for interspecific differences in full sun Amax and Rdark, but no evidence (or weak evidence in the case of Rdark_mass_) for interspecific differences in proportional down-regulation in response to shade. The R^2^ of Model 3a is 0.86 for Amax_area_, and 0.81 for Rdark_area_. The fits of Model 3a to the data are shown in Figure 1.

**Table 1:** Comparison of models of leaf maximum net photosynthetic capacity (Amax) and dark respiration rate (Rdark) in response to light level.

norm	data	model	n	df	R^2^	aic.ncor
area	Rdark	1a	56	3	0.579	−52.79
area	Rdark	1b	56	4	0.615	−55.40
area	Rdark	1c	56	7	0.772	−77.11
area	Rdark	2a	56	4	0.619	−55.98
area	Rdark	2b	56	5	0.619	−53.61
area	Rdark	2c	56	8	0.776	−75.43
**area**	**Rdark**	**3a**	**56**	**7**	**0.802**	**−85.16**
area	Rdark	3b	56	8	0.802	−82.49
area	Rdark	3c	56	11	0.810	−75.81
area	Amax	1a	41	3	0.638	186.21
area	Amax	1b	41	4	0.644	188.00
area	Amax	1c	41	7	0.846	161.96
area	Amax	2a	41	4	0.639	188.50
area	Amax	2b	41	5	0.652	189.64
area	Amax	2c	41	8	0.848	164.45
**area**	**Amax**	**3a**	**41**	**7**	**0.863**	**156.96**
area	Amax	3b	41	8	0.863	160.03
area	Amax	3c	41	11	0.871	168.18
mass	Rdark	1a	54	3	0.206	525.11
mass	Rdark	1b	54	4	0.532	498.90
mass	Rdark	1c	54	7	0.817	455.83
mass	Rdark	2a	54	4	0.538	498.19
mass	Rdark	2b	54	5	0.539	500.51
mass	Rdark	2c	54	8	0.826	455.94
mass	Rdark	3a	54	7	0.806	458.94
mass	Rdark	3b	54	8	0.807	461.52
**mass**	**Rdark**	**3c**	**54**	**11**	**0.856**	**454.74**
mass	Amax	1a	40	3	0.246	605.10
mass	Amax	1b	40	4	0.287	605.33
mass	Amax	1c	40	7	0.683	581.26
mass	Amax	2a	40	4	0.295	604.89
mass	Amax	2b	40	5	0.302	607.09
mass	Amax	2c	40	8	0.692	583.30
**mass**	**Amax**	**3a**	**40**	**7**	**0.786**	**565.53**
mass	Amax	3b	40	8	0.788	568.32
mass	Amax	3c	40	11	0.818	573.04

The models here are the nine possible combinations of, (1) a single value of μ (i.e., full-sun Amax or Rdark) shared by all species, (2) separate μ for deciduous and conifer trees, (3) species-specific μ; and (a) a single value of D for all species, (b) separate D for deciduous and conifer species, (c) species-specific D. The data are normalized either by area or by mass (norm). The number of parameters (df) of each model includes the variance of the error term in Equation (1). R^2^ is the coefficient of determination describing the overall fit of the model to data; and the Akaike Information Criterion (aic.ncor) is sample size-corrected following Bolker [35], 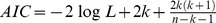, where k is the number of parameters, and n is the sample size. The AIC index without sample size-corrected showed the same results. The best model(s) (lowest aic.ncor) is highlighted in bold.

The maximum likelihood estimates for Model 3a are shown in Table 2. Among angiosperms, shade intolerant species (gray birch and white ash) had significantly higher full-sun values of mass- and area-based Amax and Rdark (μ) than the shade tolerant species (sugar maple). In contrast, for conifers, there was no significant difference in μ between shade intolerant (eastern white pine) and shade tolerant species (eastern hemlock). Gray birch had significantly higher full-sun per-area and per-mass values of Amax than all other species.

**Table 2:** Maximum likelihood estimates (MLE) and 95% confidence limits for parameters in Model 3a.

		Amax_area_	Amax_mass_	Rdark_area_	Rdark_mass_
D	lower limit	0.325	−0.653	0.761	0.170
	MLE	0.554	−0.037	0.849	0.425
	upper limit	0.726	0.324	0.916	0.597
μ_birch	lower limit	13.52	1238.0	0.98	88.8
	MLE	16.00	1700.0	1.12	109.2
	upper limit	18.44	2157.7	1.26	129.7
μ_ash	lower limit	4.21	446.6	1.26	112.3
	MLE	6.27	693.8	1.55	147.7
	upper limit	8.97	1014.8	1.87	187.2
μ_maple	lower limit	2.82	335.2	0.56	53.1
	MLE	4.98	550.3	0.75	73.3
	upper limit	7.93	838.2	0.95	96.7
μ_pine	lower limit	5.00	282.2	0.59	24.7
	MLE	7.65	497.9	0.81	39.8
	upper limit	11.55	807.1	1.06	59.2
μ_hemlock	lower limit	4.18	303.8	0.71	40.6
	MLE	6.86	544.7	1.04	62.1
	upper limit	10.67	886.0	1.45	89.9

Units for per-area Amax and Rdark are µmol CO_2_ m^−2^ s^−1^, and units for per-mass Amax and Rdark are 10^−4^ µmol CO_2_ g^−1^ s^−1^.

### 2. One can accurately predict a leaf's Amax and Rdark given the light level above the leaf and one species-specific number (either full-sun Amax or full-sun Rdark)

**Figure 2:**
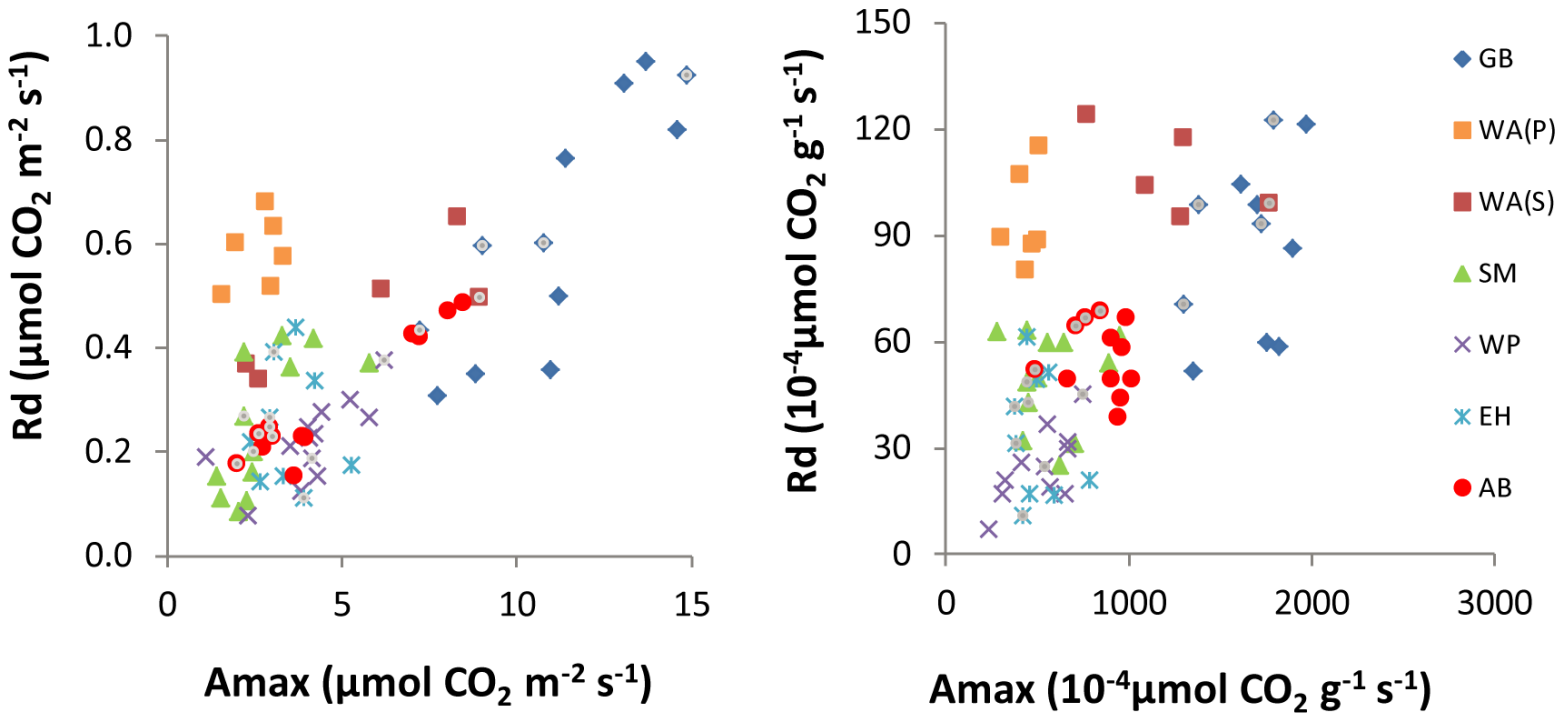
Relationships between Amax and Rdark. The left panel shows area-based rates, and the right panel shows mass-based rates. Symbols marked by small grey dots indicate shade leaves of sun grown trees. Species code: GB = gray birch, WA(P) = white ash sampled at the Princeton site, WA(S) = white ash sampled at the Stokes site, SM = sugar maple, WP = white pine, EH = eastern hemlock, AB = American beech.

Many previous papers have documented that a leaf's maximum photosynthetic capacity is approximately proportional to its dark respiration rate (e.g. Givnish [9]), and our study confirms this result (Figure 2). The area-based values (Figure 2A) show a cleaner relationship than the mass-based values (Figure 2B; correlation coefficients of 0.72 vs. 0.56). Collectively, the tight correlation in Figure 2A, Result 1, and the high R^2^ values for Model 3a in Table 1 imply that one can predict a leaf's Amax and its Rdark with considerable accuracy given the light level above the leaf and one species-specific number (either the full-sun Amax or full-sun Rdark). The correlation in Figure 2A improves to 0.86 if we remove the 6 outliers (all white ash), which were the only individuals sampled at a ridge-top location within the Princeton site. Two observations suggest that these Princeton white ash saplings suffered from drought stress: (1) they have low stomatal conductance (for a given light level) compared to white ash sampled at the Stokes field site (Figure S1); and (2) they had some withered leaves at the time of sampling, unlike saplings at other locations in our study. Note that white ash samples from the Stokes field site share the same linear Rdark-Amax relationship with the other species (Figure 2A).

### 3. Amax_area_ and Rdark_area_ are both significantly down regulated in partial sun, whereas Amax_mass_ and Rdark_mass_ are either not down regulated or weakly down regulated

The confidence limits for Model 3a in Table 2 show that D is significantly positive for area-based Amax and Rdark, meaning that both Amax_area_ and Rdark_area_ are significantly down regulated as light decreases. Pooling all models, 42 out of 48 estimates for area-based D were significantly positive.

**Figure 3:**
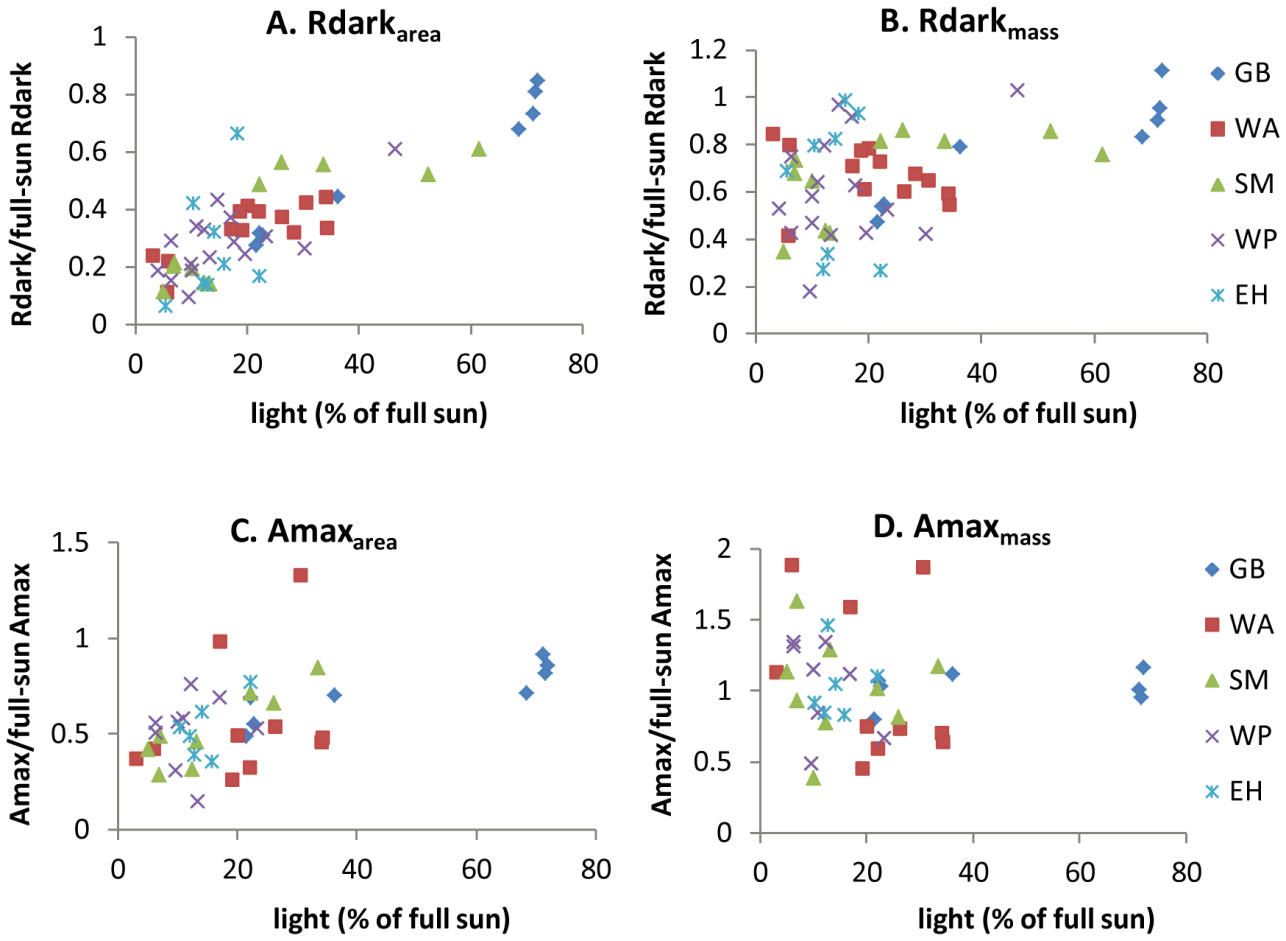
Relationship between normalized Amax or Rdark (measured value divided by species mean full-sun value) and light. Species code: GB = gray birch, WA = white ash, SM = sugar maple, WP = white pine, EH = eastern hemlock.

Dividing both sides of equation (1) by μ_i_ yields the proportional down regulation of a leaf, whose expectation (1-D +D L_ij_) has no species-specific quantities given that all species share the same value of D (Result 1). Thus, a scatter plot of Amax and Rdark normalized by their estimated μ_i_ from Model 3a against light level should allow us to observe the level of down regulation in the data without being distracted by interspecific differences in μ. Note that area-based Rdark/(Full-sun Rdark) increases linearly with light in Figure 3A and that all species appear to be on the same line, consistent with Model 3a. Area-based Amax/(Full-sun Amax) also increases with light (Figure 3C) but with more scatter than Rdark/(Full-sun Rdark), which confirms that respiration is more tightly down regulated than Amax.

In contrast to area-based values, D is not significantly different from zero for mass-based Amax in Model 3a and is only weakly significantly positive for mass-based Rdark. Pooling all models, only 9 out of 48 estimates for mass-based D were significantly positive. Weak or non-existent down regulation of mass-based Rdark and Amax is confirmed by the scatter plots in Figure 3C and 3D. Note the weak positive correlation between light and Rdark/(Full-sun Rdark) and the absence of any correlation between light and Amax/(Full-sun Amax) in Figure 3D.

### 4. Most of the down regulation of area-based Rdark and Amax is caused by reductions in LMA as light decreases. Leaves get thinner as light decreases while mass based Amax and Rdark remain approximately constant. This explains Result 3

**Figure 4:**
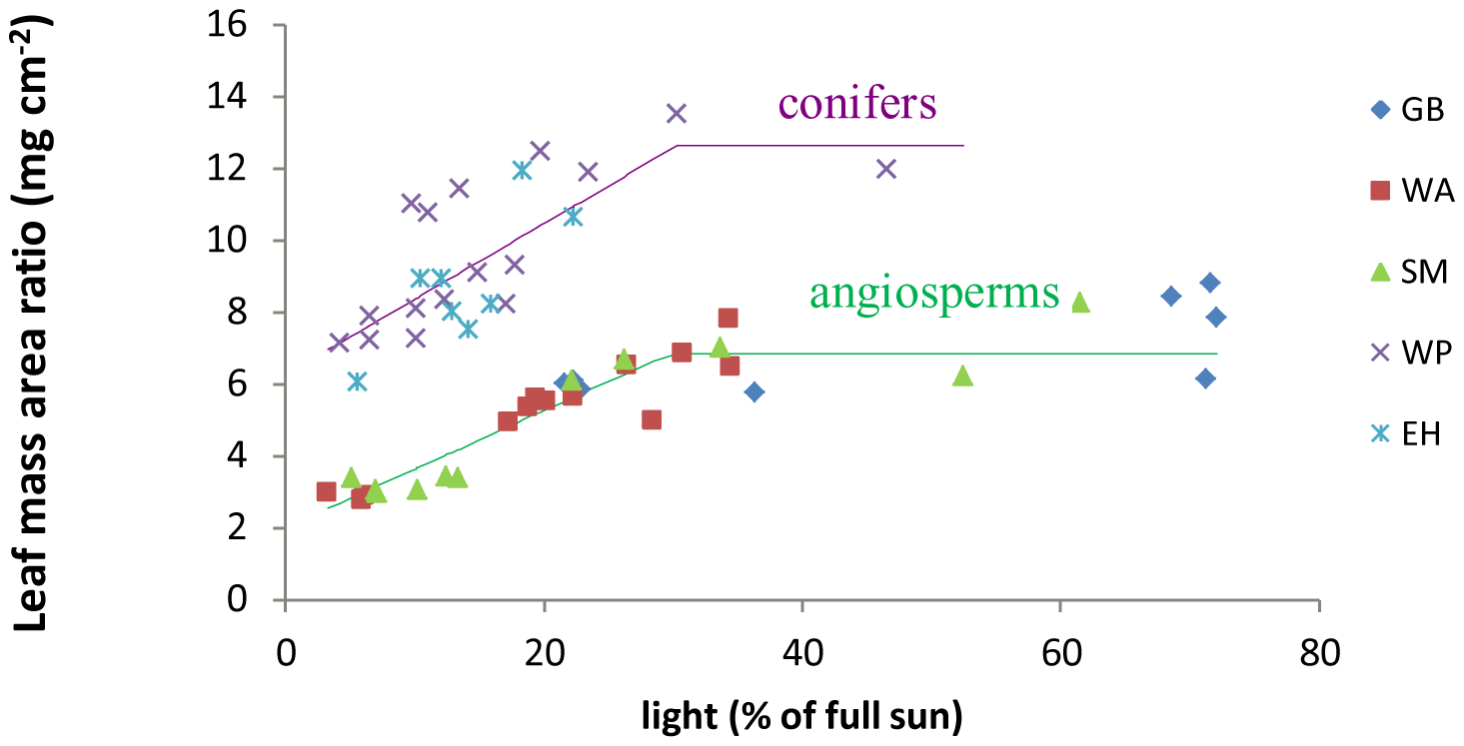
Relationship between leaf mass area ratio (LMA, mg/cm^2^) and leaf-level irradiance. For both angiosperm and conifer species, LMA can be expressed as a linear function of light (L, % of full sun) when light is less than 30%. For angiosperm species, the expression is LMA = 0.163*L+1.997 (R^2^ = 0.85, P<<0.001); and the expression for conifer species is LMA = 0.233 *L+6.044 (R^2^ = 0.45, P<<0.001). The plateau values of LMA when light is above about 30% are 6.83 mg/cm^2^ for angiosperm species, and 12.64 mg/cm^2^ for conifer species. Species code: GB = gray birch, WA = white ash, SM = sugar maple, WP = white pine, EH = eastern hemlock.

**Figure 5:**
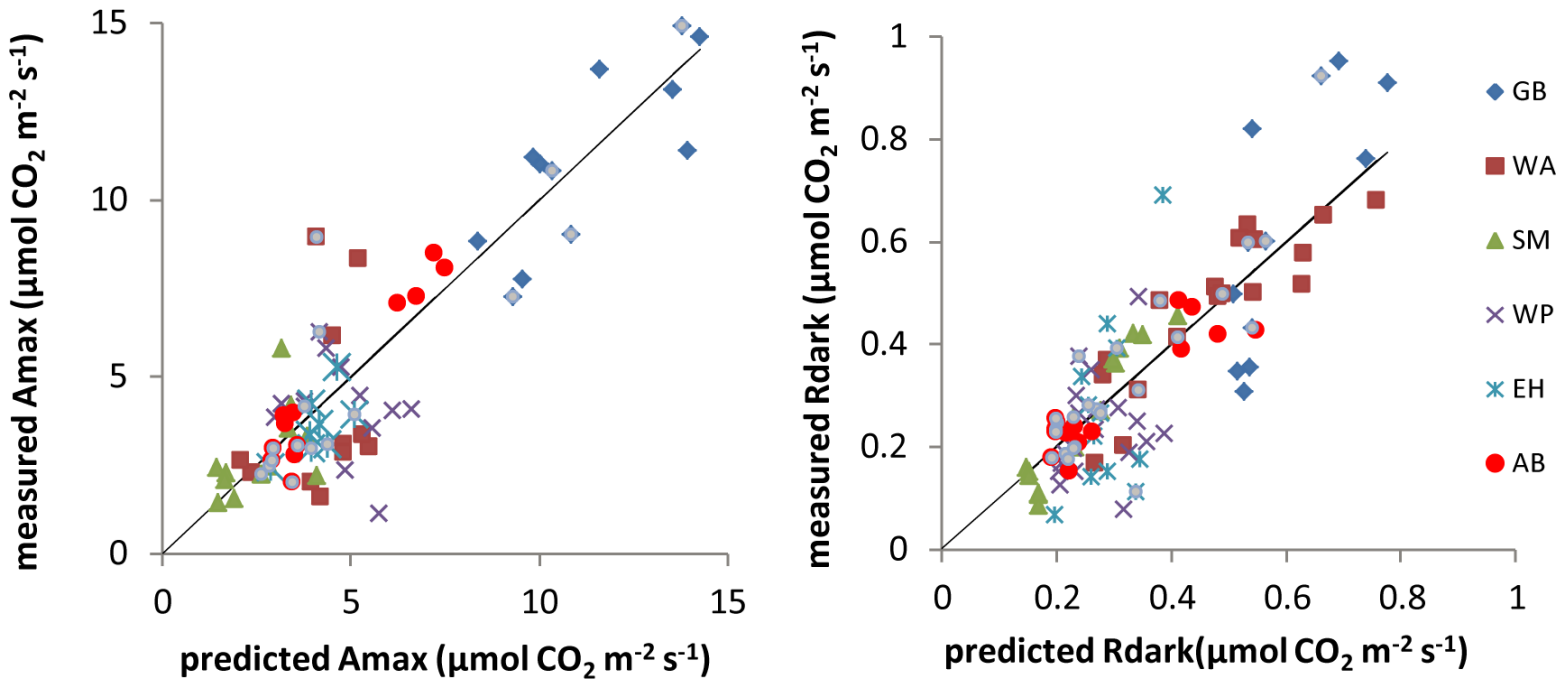
Predicted per-area leaf photosynthetic capacity (Amax_area_) and dark respiration rate (Rdark_area_) vs. observed values. The model (Equation 2 in main text) assumes that each leaf's Amax_area_ (or Rdark_area_) is equal to a species-specific constant per-mass Amax (or Rdark) times the leaf's mass:area ratio (LMA). Symbols marked by small grey dots indicate shade leaves of sun grown trees. Species code: GB = gray birch, WA = white ash, SM = sugar maple, WP = white pine, EH = eastern hemlock, AB = American beech.

The relationships between light and LMA in Figure 4 show that LMAs of all species increase to an asymptote as light increases and that the functions for conifers and broad-leaved trees are different. To quantify how much of the down regulation of the area-based quantities is due solely to reductions in LMA, we fit Equation 2 to the data and estimated C_i_ as the mean of the mass-based measurements for species-i. Predicted versus actual plots of this model show that it is remarkably accurate (Figure 5). Its R^2^ is 0.79 for Amax and 0.73 for Rdark.

### 5. Shade leaves of sun-grown plants appear to follow the same pattern of down regulation as leaves of shade-grown plants

Although we lack light measurements for shade leaves of sun-grown plants, other quantities provide some information about their patterns of down regulation. For example, Figure 5 includes the samples for shade leaves of sun-grown plants (plotting symbols marked with small grey dots in Figure 5) and Equation 2 appears to work as well for them as for the other leaves. Figure 2 also includes the shade leaves of sun-grown plants, and they too appear to follow approximately the same relationship as the other leaves.

### 6. Statistical power to detect species differences in area-based down regulation is high for Rdark, but not for Amax

If the true model for area-based Rdark and Amax is Model 3c (species differ in both full-sun rates and down-regulation capacity, D), and if the true coefficient of variation (CV) in D among species is 20%, then the power of our analysis (i.e., the probability that our analysis would correctly identify Model 3c as the best model, as opposed to Model 3a, in which species are assumed to differ in full-sun rates but not in D) is 0.77 for Rdark but only 0.22 for Amax (Figure S2). If the true CV in D among species is 30%, then the power of our analysis increases to 0.91 for Rdark and 0.32 for Amax. If we instead quantify the level of interspecific difference in D relative to the level of interspecific difference in full-sun rates (μ in Equation 1) by the ratio of interspecific CV in D to interspecific CV in μ (CV_D_:CV_μ_), we obtain the same qualitative result of relatively high power for Rdark and relatively low power for Amax. For example, for CV_D_:CV_μ_ values of 0.7 and 1.0, respectively, the power of our analysis is about 0.77 and 0.91 for Rdark and about 0.32 and 0.43 for Amax (Figure S3). In summary, given the level of random error in the data, our sample design (which includes both sample size and the range of light values sampled for each species) has relatively high power to detect at least modest interspecific differences in D for Rdark, but not for Amax.

Doubling our sample size would yield high power to detect small interspecific differences in D (e.g., CV_D_:CV_μ_ = 0.5; Figure S3) for Rdark but not for Amax. Power to detect species differences in D for Amax is sensitive to the distribution of sampled light levels (compare power for observed vs. random uniform light levels in Figures S2 and S3; see Figure S4 for the distribution of sampled light levels). For Amax, high power to detect small interspecific differences in D would require either a dramatic increase in sample size or a more balanced distribution of sampled light levels (i.e., more high-light measurements; see Figure S4).

## Discussion

Our analysis of a novel dataset, including leaves from saplings of six temperate tree species spanning the full range of light conditions under which each species occurs, suggests a species-independent trajectory of Amax and Rdark down-regulation in response to shade. Although species have different high-light Amax and Rdark values (Table 2), leaves of all species appear to follow the same down-regulatory path in terms of their proportional decrease in Amax and Rdark with decreasing whole-plant light availability. Furthermore, shade leaves of sun-grown saplings appear to lie on the same down-regulatory pathway as leaves of shaded saplings. Although we did not measure leaf-level irradiances for shade leaves of sun-grown saplings, the fact that they showed the same relationships between Amax, Rdark, and LMA as other leaves (Figures 2 and 5) suggests a common regulatory pathway.

For Rdark, our failure to detect significant interspecific differences in down-regulation, combined with high statistical power to detect at least modest differences (Figures S2, S3), suggests that any such differences that actually occur are small. In contrast, our analysis had low power to detect interspecific differences in Amax down-regulation, so our failure to detect such differences should be interpreted with caution. Nevertheless, our well-supported conclusion that interspecific differences in Rdark regulation are likely to be small, combined with the well-known correlation between Amax and Rdark (Figure 2; ref [9]), suggests that interspecific differences in Amax down-regulation are also likely to be small. A more powerful sampling design (including both larger sample sizes and a more even distribution of sampled light levels; Figures S2, S3, S4) would be a useful next step towards quantifying the degree of similarity/difference among species in Amax down regulation.

Our results suggest a simple rule for Amax and Rdark down-regulation that could be implemented in forest carbon cycle models to calculate Amax and Rdark (either mass- or area-based) of individual leaves or leaf layers. Specifically, given only the light level above a leaf and one species-specific number (either the full-sun Amax or full-sun Rdark), Equation 1 and the parameter values in Table 2 predict Amax and Rdark with considerable accuracy. Note that Amax and Rdark are highly correlated with each other (Figure 2; ref [9]), so that only one full-sun value (either Amax or Rdark) is needed to predict both Amax and Rdark of a given leaf.

Our findings also imply a simple mechanism of Amax and Rdark down-regulation. Down-regulation in our study and others [37]–[39] appears to be mostly area-based, not mass-based. In our study, mass-based values are roughly constant with respect to light; i.e., area-based down-regulation occurs primarily by decreasing LMA while maintaining roughly constant per-mass values. Three lines of evidence support this claim. First, Equation 2 accurately predicts Amax_area_ (or Rdark_area_) of a leaf given its LMA and a species-specific constant value of Amax_mass_ (or Rdark_mass_). Secondly, compared to shade leaves from sun-grown plants or leaves from shade-grown plants, sun leaves have consistently higher values of LMA, Amax_area_, and Rdark_area_; whereas no clear pattern emerges for Amax_mass_ and Rdark_mass_ across the three leaf types (Figures S5, S6, S7). Finally, the parameter estimates in Table 2 suggest strong area-based, but not mass-based, down-regulation in response to shade. Together, these results imply that area-based down-regulation is due primarily to combining constant mass-based rates with LMA values that decrease as light decreases.

While this simple model highlights the significant role of LMA in a tree's adaptive plasticity to an unpredictable light environment [9], [40]–[42], it nevertheless downplays the role of physiological down-regulation in response to shade suppression [9], [43]. LMA does not explain all changes in Amax and Rdark. In particular, Rdark_mass_ is significantly down-regulated with decreasing light but with a slope that is much less than the per-area slope. This small extra respiratory down-regulation makes LMA a better predictor for Amax_area_ than for Rdark_area_, particularly for saplings grown in high light (Result 4 and Figure 5). Also, although LMA saturates when light is above about 30% (Figure 4), Amax_area_ and Rdark_area_ continue to increase (Figure 3). The residual down-regulation of Amax and Rdark is likely through the changes in physiological processes, for instance, the activity of the photosynthetic enzyme Ribulose-1,5-bisphosphate carboxylase/oxygenase (RuBisCO) [43].

Our results have important implications for canopy integration schemes used to calculate photosynthesis in some global carbon cycle models. Based on optimal nitrogen allocation theory, Sellers et al. [13] predicted that maximum carboxylation capacity (Vcmax; roughly proportional to Amax_area_) of a given leaf should equal top-of-canopy Vcmax multiplied by light (proportion of top-of-canopy irradiance; see their Equation 24a). This model corresponds to a special case of our Equation 1 with unlimited down-regulation capacity (D = 1). For Amax_area_, our maximum likelihood estimate for D is 0.55, with an upper confidence limit of 0.71. This implies a substantial error in the Sellers et al. [13] scheme, which has been adopted or modified by several global carbon models (e.g., refs [44], [45]). A key point is that D<1 in our model implies that light-dependent physiological rates do not approach zero with decreasing light, as assumed by the Sellers et al. [13] model and its derivatives. In contrast to this discrepancy with some existing canopy integration schemes, our LMA-based model of down-regulation (Equation 2) is consistent with Thornton and Zimmermann's [14] model for Vcmax (their Equations 1, 6, and 7), which they assume is proportional to nitrogen concentration per unit leaf mass (assumed constant for each plant functional type) divided by specific leaf area (assumed to increase linearly with overlying leaf area). Our results imply that the approach of Thornton and Zimmermann [14] could be extended to deal with within-functional-type diversity if a single mass-based constant (e.g., Amax_mass_) were available for each species.

In addition to providing a simple description of down-regulation that could be incorporated into carbon cycle models, our finding that all species share a single down-regulatory pathway sheds light on the role of leaf physiology in maintaining species diversity. Specifically, if all species have the same ability to down-regulate (i.e., the same proportional decrease in full-sun Amax and Rdark with decreasing light) – as our results suggest – then the rank order of species' Amax and Rdark rates is the same at any light level; i.e., the species with the highest (lowest) Amax in full sun also has the highest (lowest) Rdark at all light levels. This qualitative result has been previously observed with tree seedlings under low- and high-light conditions [39], [46]. Our study generalizes the result to saplings across the full light gradient and suggests that a species-independent down-regulatory path may be a key component of the physiological successional tradeoff. This mechanism does not preclude a role for allocational or other tradeoffs in maintaining successional diversity [46]–[48].

Our main result, that leaves – regardless of species identity or whole-plant light availability – reduce Amax and Rdark by a similar fraction between high and low light environments, requires several qualifications. Firstly, we studied only six species in a single geographic region. Although the statistical power of our analysis to detect interspecific differences was high for Rdark, it was low for Amax. The low signal to noise ratio of Amax in this study is particularly evident if gray birch is excluded from the analyses (Table S2). Gray birch was largely found growing in much higher light than saplings of other species, which led to an unbalanced sampling design to detect interspecific differences. For Rdark, species-independent down-regulation is still evident for Rdark_area_ if gray birch is excluded, but not for Rdark_mass_ (Table S2). These contrasting results for Rdark_area_ verse Rdark_mass_ again confirm the finding of decreasing LMA as a primary mechanism for area-based down-regulation. Also, due to logistical constraints, we measured gas exchange on leaves of detached branches. We suspect that this had little impact on our results, but it would be useful to obtain *in situ* measurements to confirm our findings. Thus, the robustness of our main result in temperate forests, and its generality across biomes, awaits further data collection and analysis. Secondly, our conclusion that down-regulation is independent of whole-plant light availability depends on indirect evidence, because we did not measure light availability for shade leaves of sun-grown saplings. This result could be confirmed with measurements of Amax, Rdark, and irradiance on shaded leaves from sun-grown and shaded saplings. Finally, our simple models do not account for the well-known effects of water and nutrients on Amax and Rdark. In particular, stomatal conductance measurements (Figure S1) suggest that the outliers in Figure 2 for white ash from the ridge-top location are likely due to edaphic conditions that were considerably different from the other locations we sampled. Future work that encompasses a broader edaphic range and measurements of water and nutrients is needed. We hope that our study will motivate other researchers to assess the robustness of our results and to extend our data and analysis to other biomes and environmental conditions. If our simple model of down-regulation proves to be general and robust, it would deepen our understanding of plant community and ecosystem ecology and would provide a simple means to substantially improve carbon cycle models.

## Supporting Information

Figure S1 **Stomatal conductance (g_s_, mol m^−2^ s^−1^) of five temperature tree species vs. growth irradiance.** Stomatal conductance was recorded along with maximum photosynthesis capacity (Amax) using the LI-6400 system. Species code: GB = gray birch, WA(P) = white ash sampled at the Princeton site, WA(S) = white ash sampled at the Stokes site, SM = sugar maple, WP = white pine, EH = eastern hemlock, AB = American beech.
(TIF)

Figure S2 **Statistical power (y-axes: probability of detecting interspecific differences in down regulation, D) for area-based Rdark (top two rows) and Amax (bottom two rows) in relation to effect size (x-axes: coefficient of variation of D among species), sample size (columns from left to right have sample sizes that are multiples of the actual sample sizes by a factor of 1, 2, 5, 10, or 100), and the distribution of sapling light availabilities (rows: actual light values, Fig. S7, or uniformly distributed light values from zero to full sunlight).** The coefficient of variation of D quantifies the variance in species-specific D values relative to the mean value of D across all species.
(TIF)

Figure S3 **Same as Figure S2, but here the effect size (x-axes: “scaled coefficient of variation of D”) is the ratio of the coefficient of variation (CV) of D relative to the CV of species-specific mean full sun Rdark or Amax rates (μ).** The CV of μ is treated as a constant and was quantified from the maximum likelihood estimates of Model 3c fit to the actual data (the standard deviation of the species-specific estimates of μ divided by the mean of these estimates across species). Thus, Figures S2 and S3 are identical except that the CV of D values in the x-axes of Figure S2 are divided by the CV of μ to create Figure S3. This allows one to visualize the power to detect differ levels of interspecific difference in down-regulation (D) relative to the level of interspecific difference in full-sun physiological rates (μ).
(TIF)

Figure S4 **Distribution of measured light levels above the crowns of the individual saplings whose leaves were subject to Rdark and Amax measurements.** Sample size is smaller for Amax due to logistical constraints (i.e., Amax was only measured under clear-sky conditions between 10:00 a.m. and 1:00 p.m.).
(TIF)

Figure S5 **Maximum net photosynthetic capacity (Amax) per unit leaf area (a) and per unit leaf mass (b).** The bars depict mean values, and sticks are standard errors (SE). Leaf categories include: (1) upper canopy “sun” leaves from a healthy sapling grown in full sun (blue); (2) lower canopy “shade” leaves from the same sapling as in (1) (cyan); and (3) leaves from a suppressed understory sapling with very low direct and indirect light irradiance (gray). Species code: GB = gray birch, WA = white ash, SM = sugar maple, AB = American beech, WP = white pine, EH = eastern hemlock.
(TIF)

Figure S6 **Dark respiration rate (Rdark) per unit leaf area (a) and per unit leaf mass (b).** The bars depict mean values, and sticks are standard errors (SE). Leaf categories include: (1) upper canopy “sun” leaves from a healthy sapling grown in full sun (blue); (2) lower canopy “shade” leaves from the same sapling as in (1) (cyan); and (3) leaves from a suppressed understory sapling with very low direct and indirect light irradiance (gray). Species code: GB = gray birch, WA = white ash, SM = sugar maple, AB = American beech, WP = white pine, EH = eastern hemlock.
(TIF)

Figure S7 **Leaf mass area ratio (LMA).** The bars depict mean values, and sticks are standard errors (SE). Leaf categories include: (1) upper canopy “sun” leaves from a healthy sapling grown in full sun (blue); (2) lower canopy “shade” leaves from the same sapling as in (1) (cyan); and (3) leaves from a suppressed understory sapling with very low direct and indirect light irradiance (gray). Species code: GB = gray birch, WA = white ash, SM = sugar maple, AB = American beech, WP = white pine, EH = eastern hemlock.
(TIF)

Table S1 **Sample sizes of different species and measurements.** * shade leaves of sun grown trees, for which light data are not available; † no light data is available for all American beech samplings.
(DOCX)

Table S2 **Comparison of models of leaf maximum net photosynthetic capacity (Amax) and dark respiration rate (Rdark) in response to light level.** Note this table is the same as Table 1, except that the analysis presented in this table does not include data of gray birch. The symbol ‘-’ indicates that the model does not return a valid estimate of parameter μ_i_ (the mean Amax or Rdark of a species-i leaf in full sun), which needs to be positive by definition. See the description in Table 1 for more details.
(DOCX)

Text S1 (DOCX)
