# Supplementary figures and images for: Species-Independent Down-Regulation of Leaf Photosynthesis and Respiration in Response to Shading: Evidence from Six Temperate Tree Species
Source: PLoS One. 2014 Apr 11;9(4):e91798. doi: 10.1371/journal.pone.0091798 (PMC3984078; doi:10.1371/journal.pone.0091798)

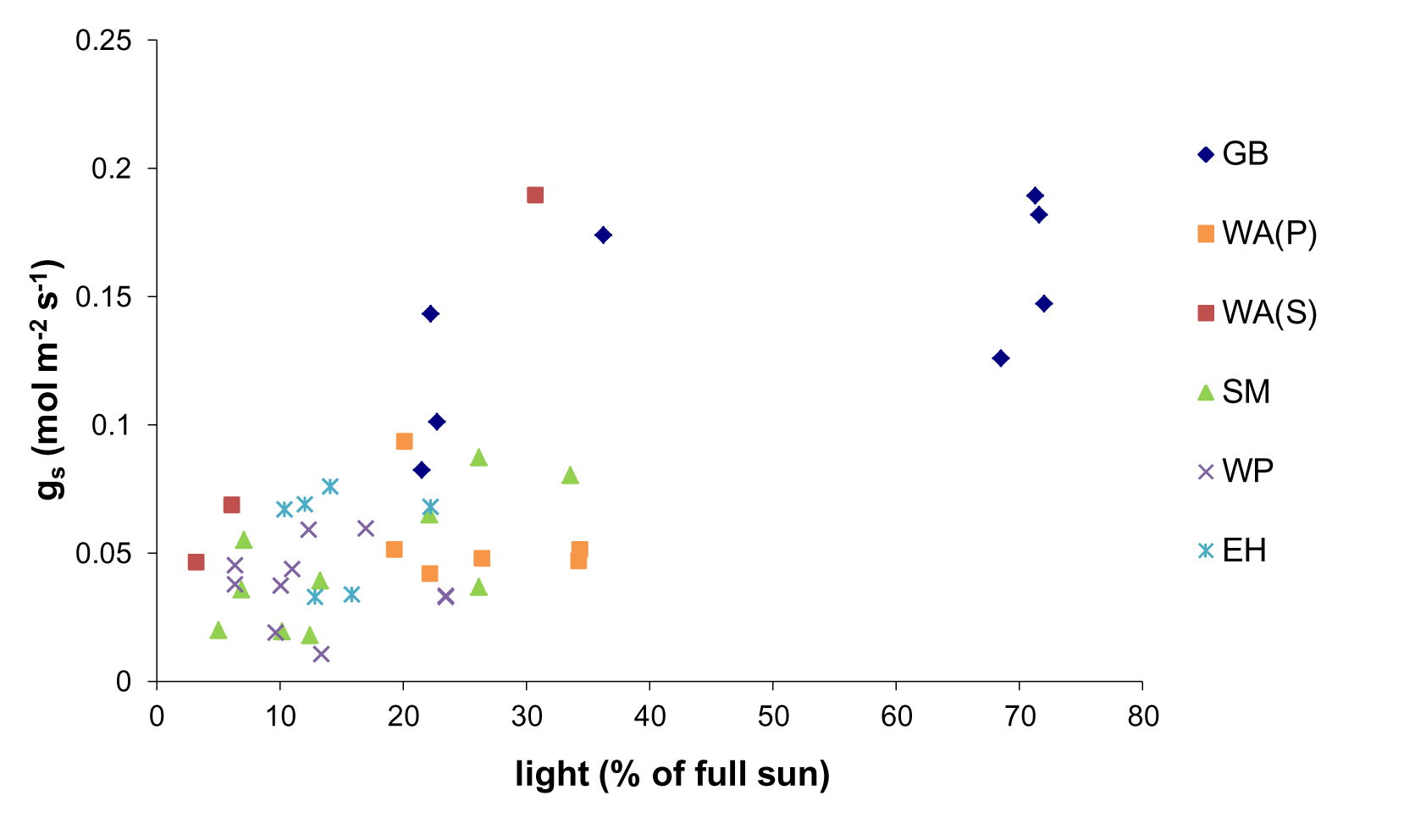

Supplement: Figure S1 — Stomatal conductance (gs, mol m−2 s−1) of five temperature tree species vs. growth irradiance. Stomatal conductance was recorded along with maximum photosynthesis capacity (Amax) using the LI-6400 system. Species code: GB = gray birch, WA(P) = white ash sampled at the Princeton site, WA(S) = white ash sampled at the Stokes site, SM = sugar maple, WP = white pine, EH = eastern hemlock, AB = American beech. (TIF) [file pone.0091798.s001.tif]

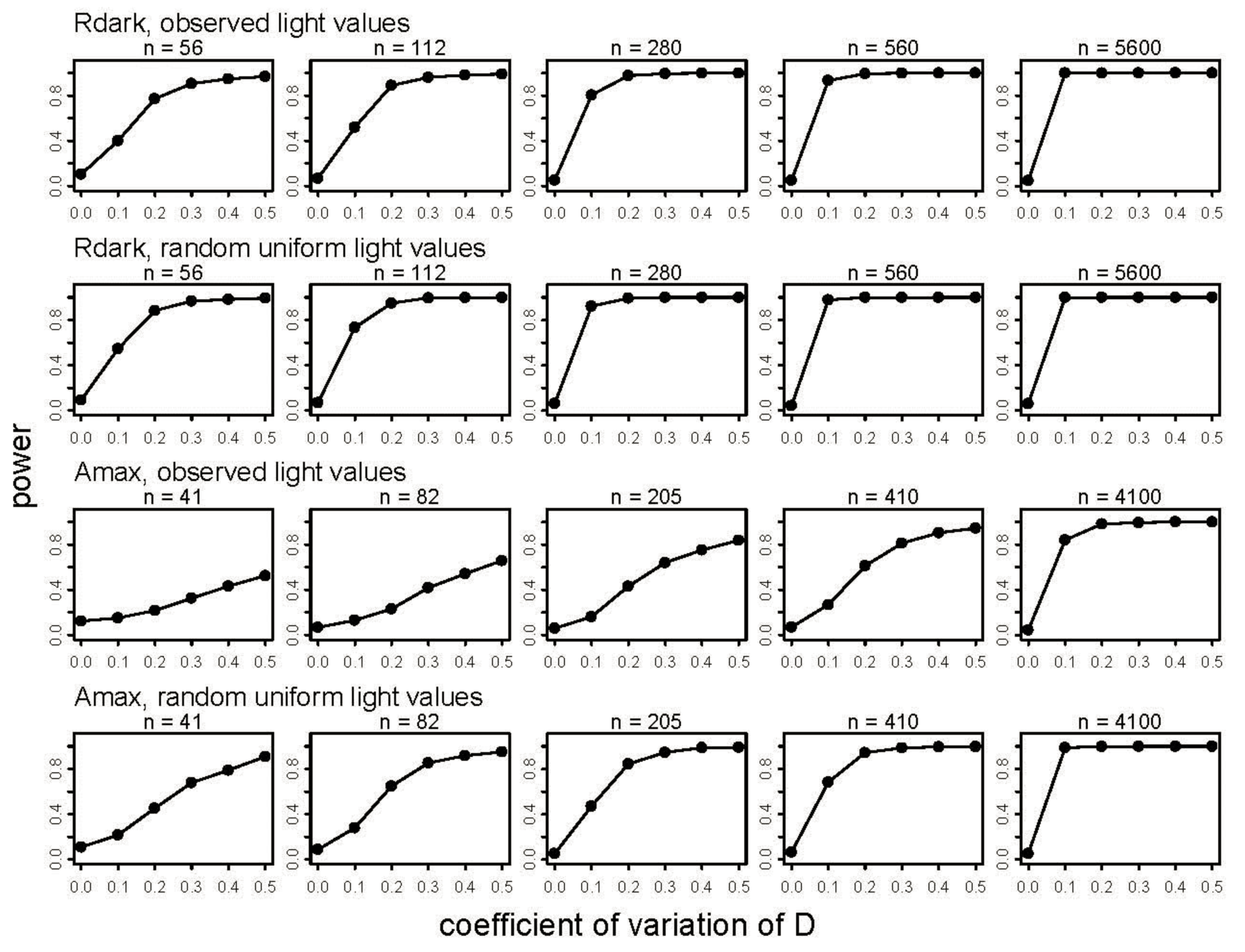

Supplement: Figure S2 — Statistical power (y-axes: probability of detecting interspecific differences in down regulation, D) for area-based Rdark (top two rows) and Amax (bottom two rows) in relation to effect size (x-axes: coefficient of variation of D among species), sample size (columns from left to right have sample sizes that are multiples of the actual sample sizes by a factor of 1, 2, 5, 10, or 100), and the distribution of sapling light availabilities (rows: actual light values, Fig. S7, or uniformly distributed light values from zero to full sunlight). The coefficient of variation of D quantifies the variance in species-specific D values relative to the mean value of D across all species. (TIF) [file pone.0091798.s002.tif]

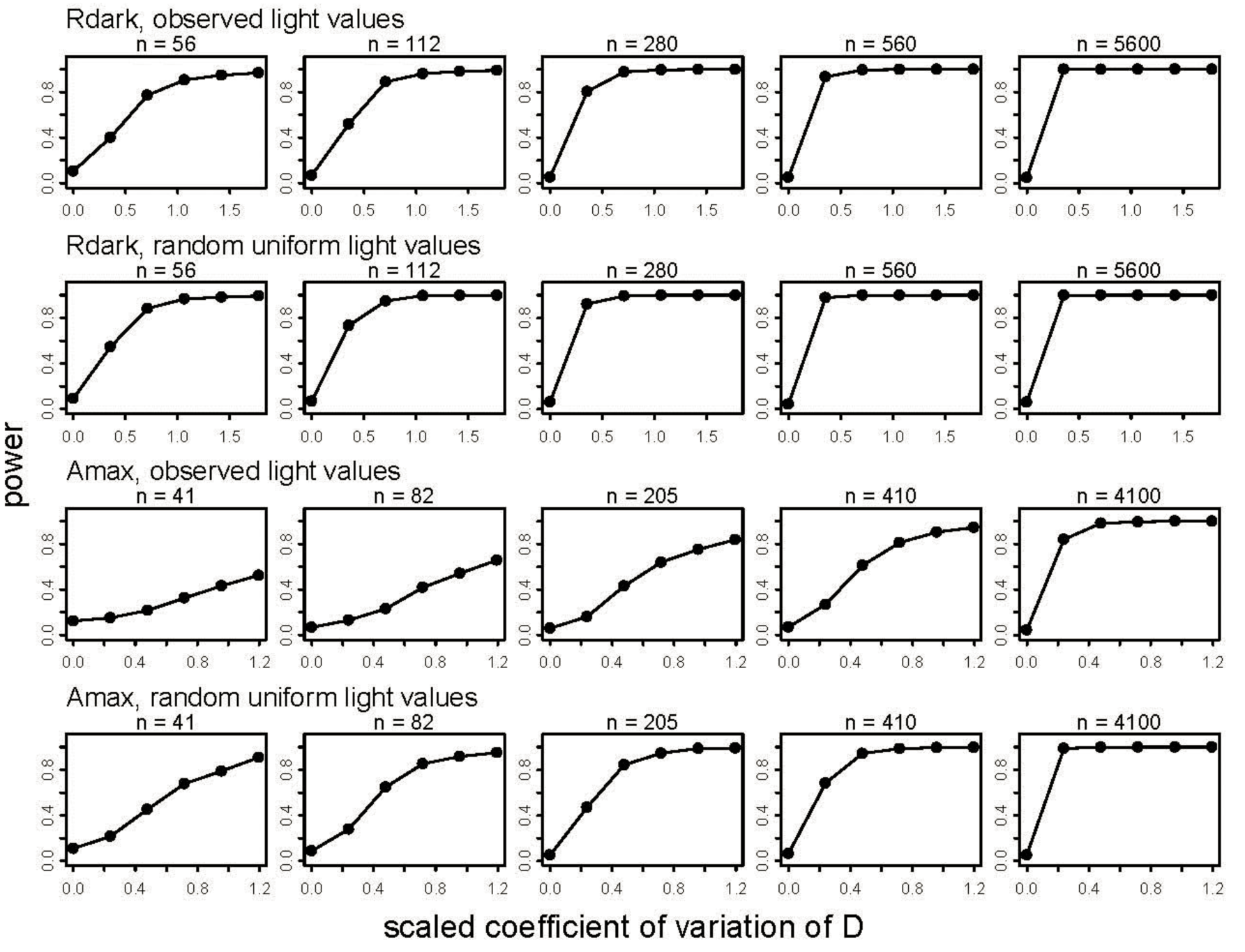

Supplement: Figure S3 — Same as Figure S2, but here the effect size (x-axes: “scaled coefficient of variation of D”) is the ratio of the coefficient of variation (CV) of D relative to the CV of species-specific mean full sun Rdark or Amax rates (μ). The CV of μ is treated as a constant and was quantified from the maximum likelihood estimates of Model 3c fit to the actual data (the standard deviation of the species-specific estimates of μ divided by the mean of these estimates across species). Thus, Figures S2 and S3 are identical except that the CV of D values in the x-axes of Figure S2 are divided by the CV of μ to create Figure S3. This allows one to visualize the power to detect differ levels of interspecific difference in down-regulation (D) relative to the level of interspecific difference in full-sun physiological rates (μ). (TIF) [file pone.0091798.s003.tif]

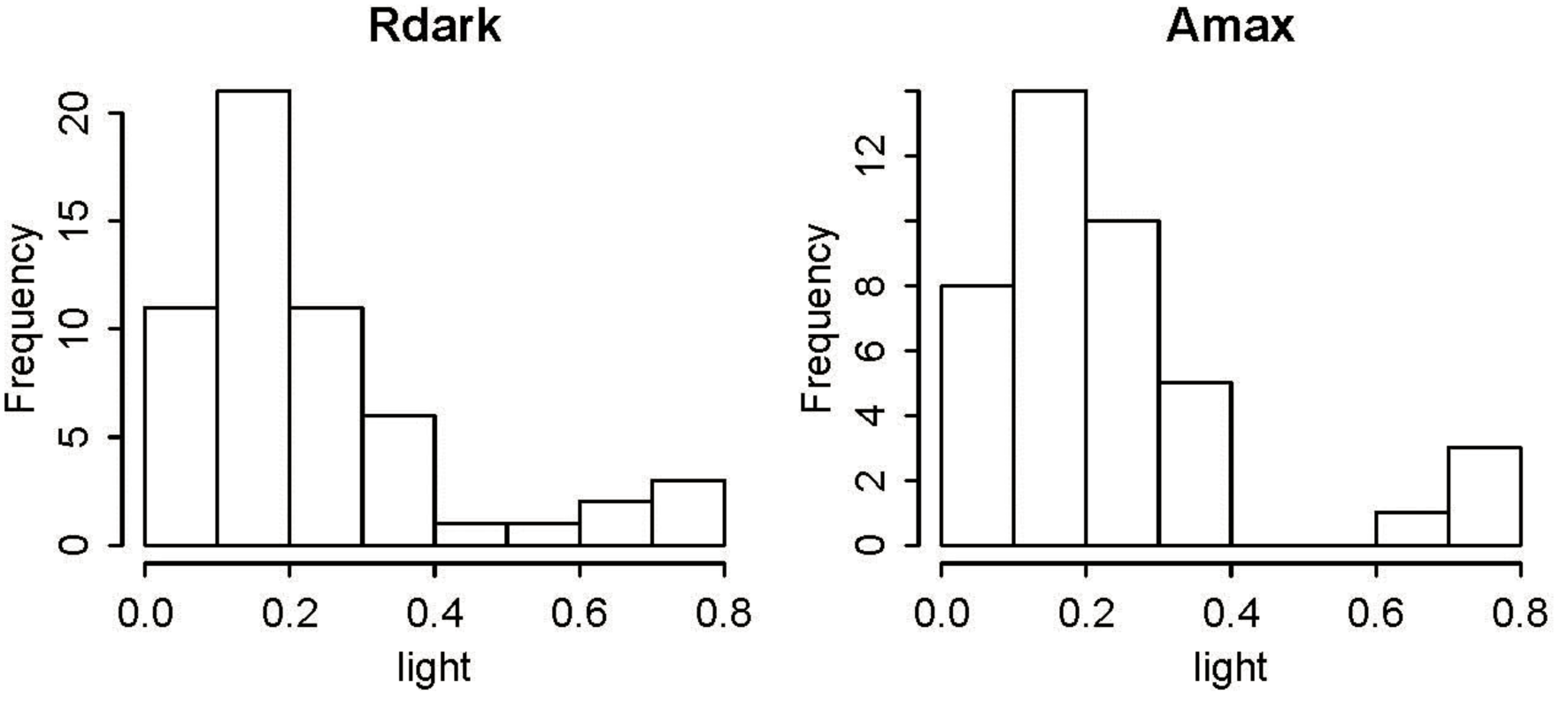

Supplement: Figure S4 — Distribution of measured light levels above the crowns of the individual saplings whose leaves were subject to Rdark and Amax measurements. Sample size is smaller for Amax due to logistical constraints (i.e., Amax was only measured under clear-sky conditions between 10:00 a.m. and 1:00 p.m.). (TIF) [file pone.0091798.s004.tif]

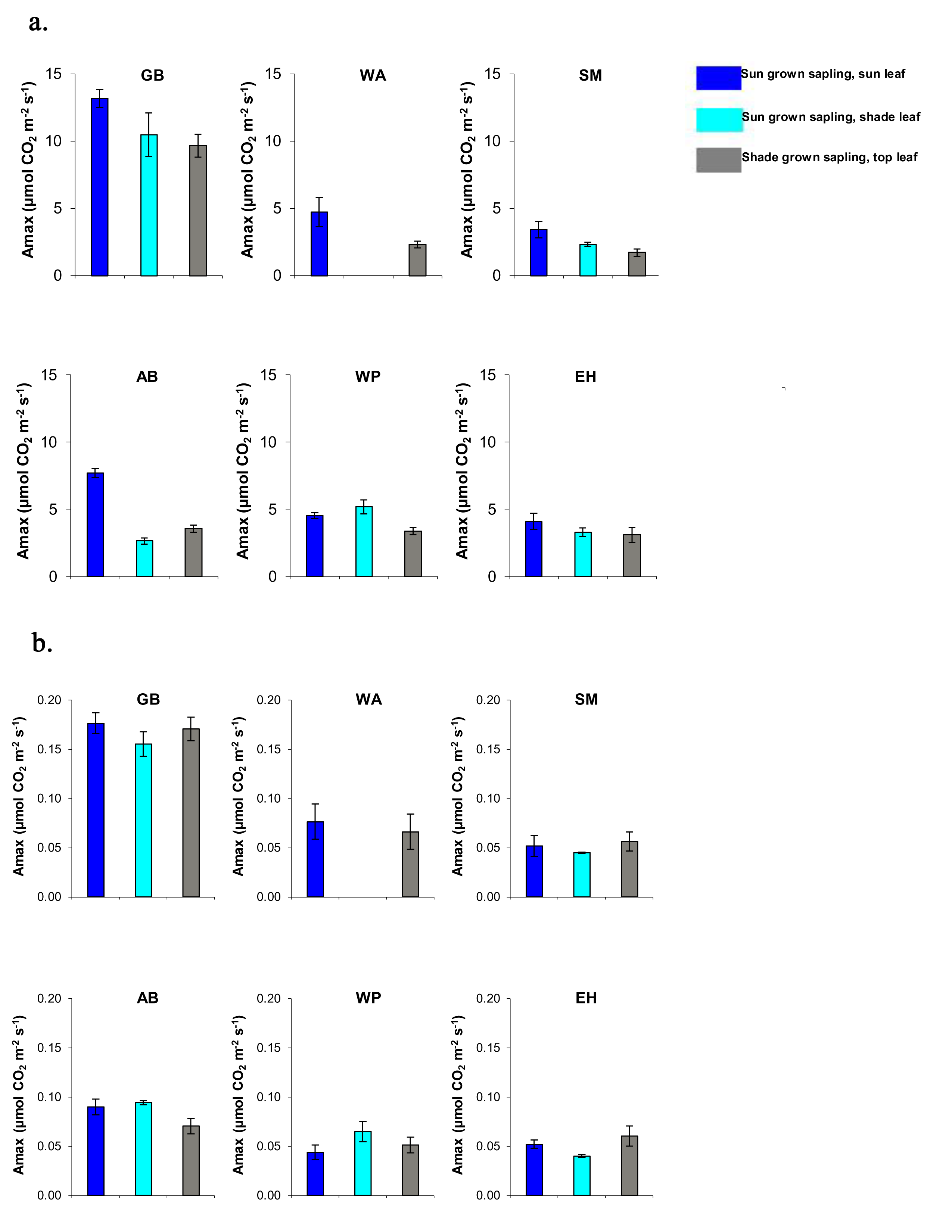

Supplement: Figure S5 — Maximum net photosynthetic capacity (Amax) per unit leaf area (a) and per unit leaf mass (b). The bars depict mean values, and sticks are standard errors (SE). Leaf categories include: (1) upper canopy “sun” leaves from a healthy sapling grown in full sun (blue); (2) lower canopy “shade” leaves from the same sapling as in (1) (cyan); and (3) leaves from a suppressed understory sapling with very low direct and indirect light irradiance (gray). Species code: GB = gray birch, WA = white ash, SM = sugar maple, AB = American beech, WP = white pine, EH = eastern hemlock. (TIF) [file pone.0091798.s005.tif]

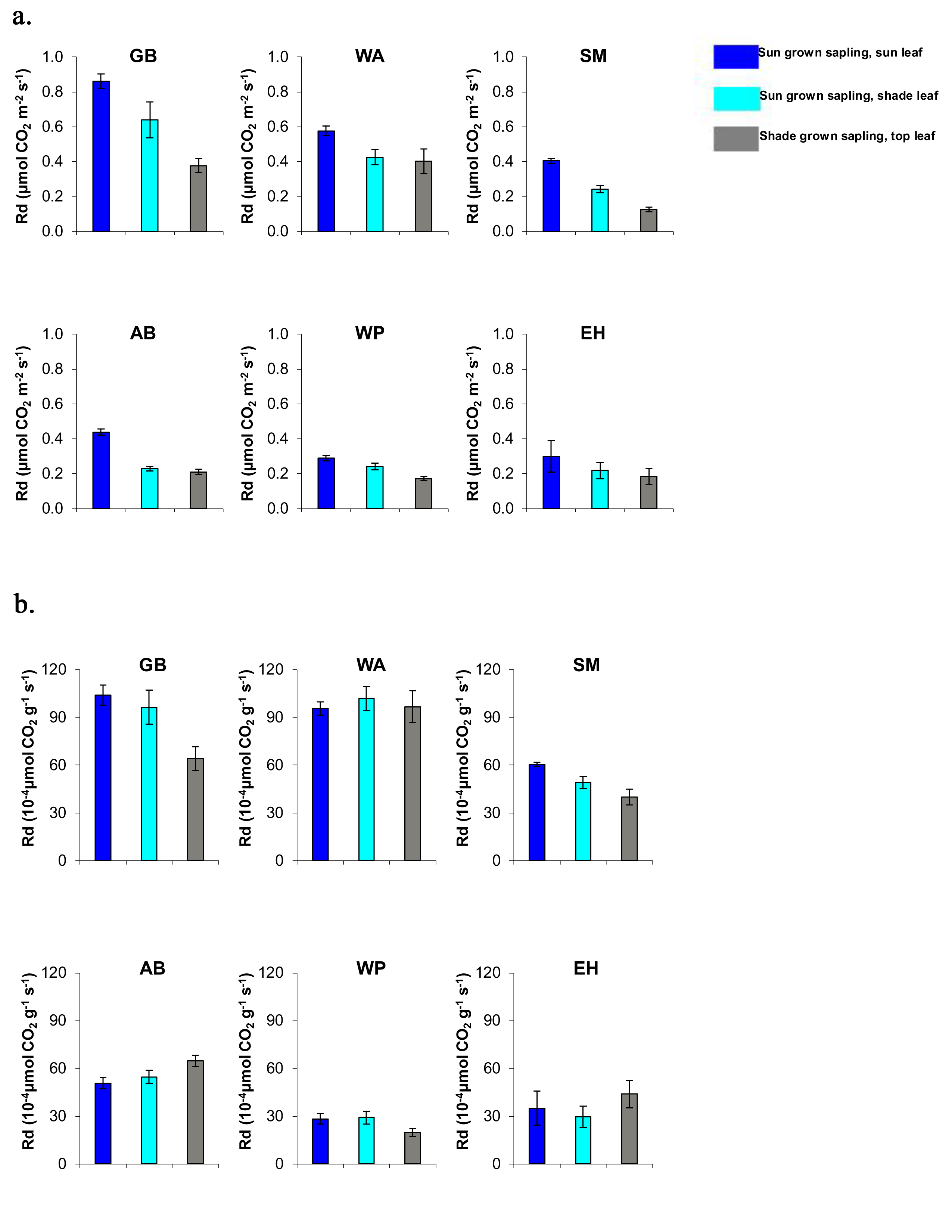

Supplement: Figure S6 — Dark respiration rate (Rdark) per unit leaf area (a) and per unit leaf mass (b). The bars depict mean values, and sticks are standard errors (SE). Leaf categories include: (1) upper canopy “sun” leaves from a healthy sapling grown in full sun (blue); (2) lower canopy “shade” leaves from the same sapling as in (1) (cyan); and (3) leaves from a suppressed understory sapling with very low direct and indirect light irradiance (gray). Species code: GB = gray birch, WA = white ash, SM = sugar maple, AB = American beech, WP = white pine, EH = eastern hemlock. (TIF) [file pone.0091798.s006.tif]

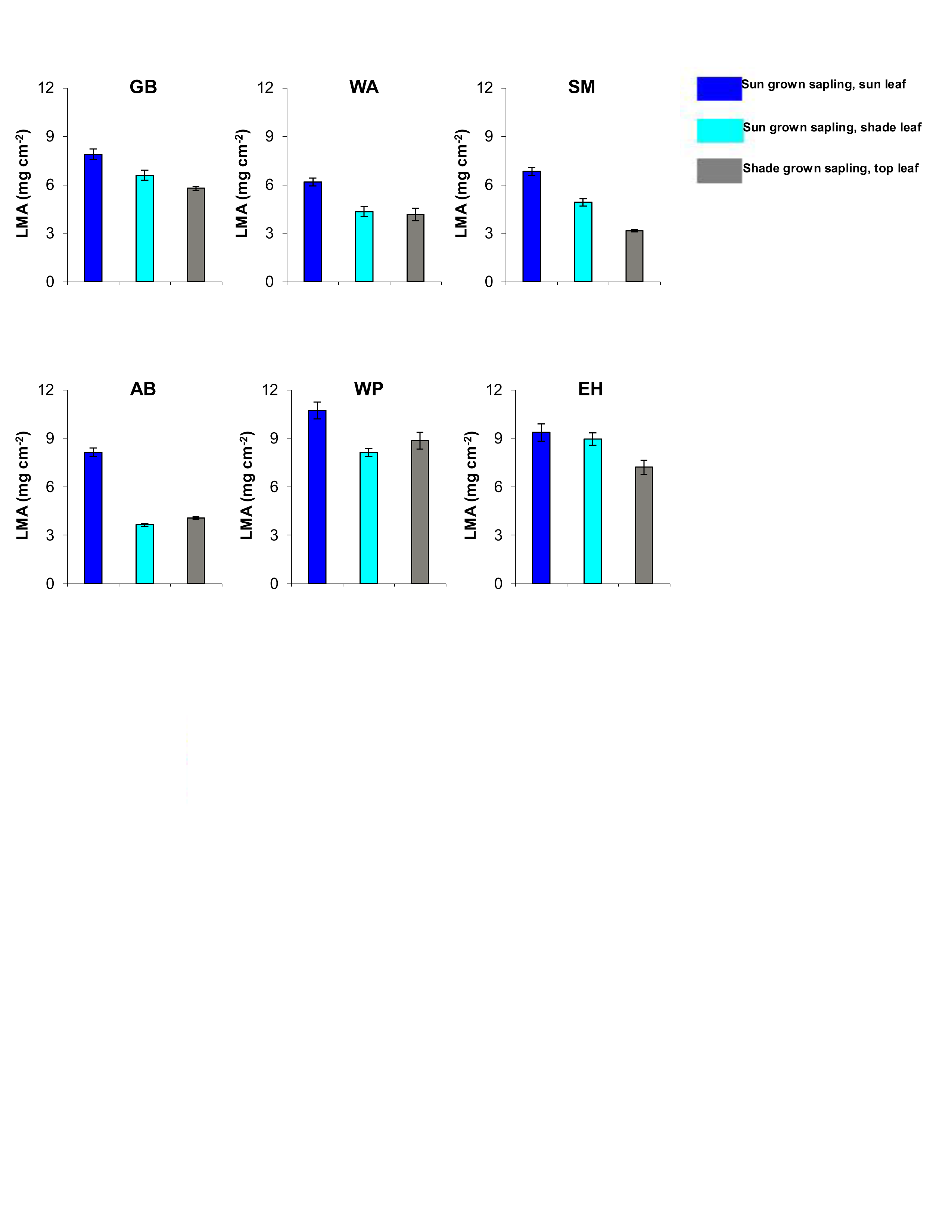

Supplement: Figure S7 — Leaf mass area ratio (LMA). The bars depict mean values, and sticks are standard errors (SE). Leaf categories include: (1) upper canopy “sun” leaves from a healthy sapling grown in full sun (blue); (2) lower canopy “shade” leaves from the same sapling as in (1) (cyan); and (3) leaves from a suppressed understory sapling with very low direct and indirect light irradiance (gray). Species code: GB = gray birch, WA = white ash, SM = sugar maple, AB = American beech, WP = white pine, EH = eastern hemlock. (TIF) [file pone.0091798.s007.tif]
