# Supplementary material for: Species-Independent Down-Regulation of Leaf Photosynthesis and Respiration in Response to Shading: Evidence from Six Temperate Tree Species
Source: PLoS One. 2014 Apr 11;9(4):e91798. doi: 10.1371/journal.pone.0091798 (PMC3984078; doi:10.1371/journal.pone.0091798)
Supplement: Table S1 — Sample sizes of different species and measurements. * shade leaves of sun grown trees, for which light data are not available; † no light data is available for all American beech samplings. (DOCX) [file pone.0091798.s008.docx]

| **species** | **data** | **n** | **additional sampling*** | |
| --- | --- | --- | --- | --- |
| gray birch | Rdark | 8 | 4 |  |
| white ash | Rdark | 13 | 4 |  |
| sugar maple | Rdark | 11 | 3 |  |
| white pine | Rdark | 16 | 5 |  |
| hemlock | Rdark | 8 | 4 |  |
| American beech† | Rdark | 10 | 5 |  |
| gray birch | Amax | 8 | 4 |  |
| white ash | Amax | 10 | 1 |  |
| sugar maple | Amax | 8 | 2 |  |
| white pine | Amax | 9 | 2 |  |
| hemlock | Amax | 6 | 3 |  |
| American beech† | Amax | 8 | 4 |  |
