# Supplementary material for: Species-Independent Down-Regulation of Leaf Photosynthesis and Respiration in Response to Shading: Evidence from Six Temperate Tree Species
Source: PLoS One. 2014 Apr 11;9(4):e91798. doi: 10.1371/journal.pone.0091798 (PMC3984078; doi:10.1371/journal.pone.0091798)
Supplement: Table S2 — Comparison of models of leaf maximum net photosynthetic capacity (Amax) and dark respiration rate (Rdark) in response to light level. Note this table is the same as Table 1, except that the analysis presented in this table does not include data of gray birch. The symbol ‘-’ indicates that the model does not return a valid estimate of parameter μi (the mean Amax or Rdark of a species-i leaf in full sun), which needs to be positive by definition. See the description in Table 1 for more details. (DOCX) [file pone.0091798.s009.docx]

| **norm** | **data** | **model** | **n** | **df** | **R^2^** | **aic.ncor** |
| --- | --- | --- | --- | --- | --- | --- |
| area | Rdark | 1a | 48 | 3 | 0.306 | -38.53 |
| area | Rdark | 1b | 48 | 4 | 0.379 | -41.50 |
| area | Rdark | 1c | 48 | 6 | 0.647 | -63.45 |
| area | Rdark | 2a | 48 | 4 | 0.376 | -41.26 |
| area | Rdark | 2b | 48 | 5 | 0.380 | -39.09 |
| area | Rdark | 2c | 48 | 7 | 0.647 | -60.76 |
| **area** | **Rdark** | **3a** | **48** | **6** | **0.678** | **-67.90** |
| area | Rdark | 3b | 48 | 7 | 0.680 | -65.41 |
| area | Rdark | 3c | 48 | 9 | 0.685 | -60.35 |
| area | Amax | 1a | 33 | 3 | 0.083 | 125.11 |
| **area** | **Amax** | **1b** | **33** | **4** | **0.200** | **123.22** |
| area | Amax | 1c | 33 | 6 | 0.230 | 127.76 |
| area | Amax | 2a | 33 | 4 | 0.190 | 123.61 |
| area | Amax | 2b | 33 | 5 | 0.200 | 125.98 |
| area | Amax | 2c | 33 | 7 | 0.230 | 131.01 |
| area | Amax | 3a | 33 | 6 | 0.216 | 128.34 |
| area | Amax | 3b | 33 | 7 | 0.221 | 131.39 |
| area | Amax | 3c | 33 | 9 | 0.244 | 137.71 |
| mass | Rdark | 1a | 46 | 3 | 0.060 | 454.61 |
| mass | Rdark | 1b | 46 | 4 | 0.492 | 428.66 |
| **mass** | **Rdark** | **1c** | **46** | **6** | **0.835** | **382.25** |
| mass | Rdark | 2a | 46 | 4 | 0.490 | 428.89 |
| mass | Rdark | 2b | 46 | 5 | 0.493 | 431.08 |
| mass | Rdark | 2c | 46 | 7 | 0.835 | 384.82 |
| mass | Rdark | 3a | 46 | 6 | 0.812 | 388.23 |
| mass | Rdark | 3b | 46 | 7 | 0.815 | 390.20 |
| mass | Rdark | 3c | 46 | 9 | 0.839 | 389.96 |
| mass | Amax | 1a | 33 | 3 | 0.056 | 462.69 |
| mass | Amax | 1b | 33 | 4 | - | - |
| mass | Amax | 1c | 33 | 6 | - | - |
| mass | Amax | 2a | 33 | 4 | 0.047 | 465.61 |
| mass | Amax | 2b | 33 | 5 | - | - |
| mass | Amax | 2c | 33 | 7 | - | - |
| **mass** | **Amax** | **3a** | 33 | 6 | **0.186** | **460.41** |
| mass | Amax | 3b | 33 | 7 | - | - |
| mass | Amax | 3c | 33 | 9 | - | - |
