# Supplementary material for: Species-Independent Down-Regulation of Leaf Photosynthesis and Respiration in Response to Shading: Evidence from Six Temperate Tree Species
Source: PLoS One. 2014 Apr 11;9(4):e91798. doi: 10.1371/journal.pone.0091798 (PMC3984078; doi:10.1371/journal.pone.0091798)
Supplement: Text S1 — (DOCX) [file pone.0091798.s010.docx]

**Supporting Text**

Amax

Measurements of net photosynthetic capacity (Amax) per unit leaf area (Amax_area_) and per unit leaf mass (Amax_mass_) are shown in Figure S5 for sun and shade leaves of sun grown saplings and leaves of suppressed saplings. Within angiosperm species, shade intolerant species (gray birch and white ash) have a higher Amax than tolerant species (sugar maple and American beech; P<0.05 for F test comparing either intolerant species to either tolerant species based on any of the three leaf categories). For conifers, no species difference is found for either Amax_area_ (F=1.408, P=0.249) or Amax_mass_ (F=0.005, P=0.9).

For angiosperm species, shade leaves of sun grown plants generally have lower Amax_area_ than conspecific sun leaves but higher than conspecific suppressed leaves (Figure S5a). For example, Amax_area_ of shade leaves in a sun grown sugar maple sapling is slightly higher than those of suppressed saplings but slightly lower than those of sun leaves. For American beeches, shade leaves of sun grown saplings are significantly lower in Amax_area_ than sun leaves (F=152.9, P<<0.001). Yet, Amax_mass_ of shade leaves in a sun grown sapling is not significantly different from that of sun or suppressed leaves (Figure S5b). Thus, Amax_mass_ is conserved both within an individual (no difference between sun and shade leaves of sun grown individuals) and among different individuals of the same species across light environments (no difference between healthy and suppressed individuals) but differs from species to species.

Rdark

Variations within and among species in dark respiration rates mirror the patterns in Amax (Figure S6). Angiosperm species generally have a higher per-area or per-mass Rdark than coniferous species, and the respiration rate of gray birch is the highest among all species. For example, for sun leaves of a sun grown plant, Rdark_area_ of gray birch is 0.86 (±0.04) (mean value ± standard error, same expression for all others hereafter) μmol CO_2_ m^-2^ s^-1^, which is over two times of that of sugar maple (0.40±0.01 μmol CO_2_ m^-2^ s^-1^) and about three times of that of white pine (0.29±0.02 μmol CO_2_ m^-2^ s^-1^) and eastern hemlock (0.30±0.09 μmol CO_2_ m^-2^ s^-1^) (Figure S6a). For angiosperm species, shade intolerant species have a higher per-area or per-mass Rdark than shade tolerant species (Figure S6). For example, for leaves of a shade suppressed sapling, Rdark_area_ of gray birch (0.38±0.04 μmol CO_2_ m^-2^ s^-1^) is significantly higher than that of American beech (0.21 μmol CO_2_ m^-2^ s^-1^) (F=14.13, P=0.004). No difference between the two conifer species was detected in either per-area or per-mass Rdark (Figure S6, P>0.1 for both cases), except that suppressed white pine needles are significantly lower in Rdark_mass_ than suppressed eastern hemlock (F=12.8, P=0.005) (Figure S6b).

Rdark_area_ of shade leaves are generally lower than conspecific sun leaves but higher than conspecific suppressed leaves for broadleaf trees (Figure S6a); yet no statistical difference among the three leave groups in Rdark_area_ is detected for conifers (F=1.78, P=0.18). For example, Rdark_area_ of shade leaves in a healthy sugar maple sapling (0.242±0.02 μmol CO_2_ m^-2^ s^-1^) is higher than those of suppressed saplings (0.126±0.01 μmol CO_2_ m^-2^ s^-1^) but slightly lower than those of sun leaves (0.403±0.01 μmol CO_2_ m^-2^ s^-1^) (P<0.01 for any two-group comparison with F test). In contrast, values of Rdark_mass_ are approximately the same across all three categories of leaves for any species (F=1.02, P=0.36) (Figure S6b). Therefore similar to the Amax results, Rdark_mass_ is conserved both within an individual and among different individuals of the same species across light environments but differs from species to species.

LMA

Like the area-based Amax and Rdark quantities, LMA values for shaded leaves of sun-grown plants were generally between those of sun leaves and leaves of suppressed saplings except for eastern white pine and American beech (Figure S7). For example, LMA of sugar maple shade leaves (4.92±0. 21 mg cm^-2^) is significantly lower than that of sun leaves (6.84±0. 23 mg cm^-2^) (F=29.3, P<<0.0001) but significantly higher than that of suppressed leaves (3.18±0.07 mg cm^-2^) (F=99.1, P<<0.0001).

Power analysis methods

Our analysis of Equation 1 suggested that the overall best model was Model 3a, which assumes species-specific high-light rates (μ) but a single value of down-regulation (D) shared by all species. In reality, we would expect species to differ in D, but such differences may be small enough to safely ignore in many contexts. It is important to understand if our failure to detect significant interspecific differences in D is due to any such differences being small vs. the statistical power (probability of detecting an effect) of our analysis being low. We used a simulation approach to quantify statistical power to detect species differences in D. The true underlying model in these simulations was Model 3c (species-specific values of both μ and D). Thus, we define power as the probability of correctly identifying Model 3c, as opposed to Model 3a, as the best model. We quantified power in regards to three variables: the degree of interspecific differences in D, the sample size, and the distribution of sampled light levels (either the actual sampled distributions, Figure S4, or a uniform distribution between zero and full sunlight). We restricted the power analysis to down regulation of area-based values of Rdark and Amax, because mass-based down regulation was relatively small (Table 2).

We performed a factorial design in which we considered six levels of interspecific differences in D (coefficient of variation of D among species CV_D_ = 0, 0.1, 0.2, 0.3, 0.4, and 0.5), five sample sizes (actual size of our sample multiplied by 1, 2, 5, 10, and 100), and two light distributions (actual distribution sampled vs. random uniform). Thus, for each of Rdark and Amax, we considered 60 different experiments, each of which was replicated 1000 times to quantify the probability of correctly identifying Model 3c as the best model (except when CV_D_ = 0, in which case Model 3a is the correct model). Each of these 60,000 realizations of the model (60 experiments × 1000 replicates) entailed (i) creating a randomly generated dataset, (ii) fitting Models 3a and 3c to the randomly generated data, and (iii) performing a likelihood ratio test to determine if Model 3c fit the data significantly better than Model 3a. We also used the Akaike Information Criterion (AIC) and the sample-size-corrected version of AIC (see notes below Table 1) to compare Models 3a and 3c. The standard AIC had an inflated Type I error rate when the sample size was small; i.e., if CV_D_ = 0, then Model 3a is in fact the true model, and Type I errors (in which Model 3c is incorrectly identified as the correct model) should occur in 5% of the replicates. In contrast, the likelihood ratio test and the sample-size-corrected AIC yielded similar results and both had Type I error rates near the nominal value of 0.05. We only report results for the likelihood ratio test.

We used the maximum likelihood estimates of D from Model 3c (applied to the actual data) to define the mean D across species, D_mean_. In each realization, we generated species-specific values of D by drawing five random numbers (one for each of the five species listed in Table 2) from a normal distribution with mean D_mean_ and standard deviation D_mean_×CV_D_. We assigned one of the randomly generated D values to each of the five species, and then generated one value x_ij_ for each individual in the experiment according to Equation 1, with a random error ε_ij_ drawn from a normal distribution with mean zero and variance equal to the Model 3c maximum likelihood estimate for the variance of ε_ij_ (i.e., when Model 3c was fit to the actual data set). Depending on the sample size in a given experiment, each species in the experiment was represented by 1, 2, 5, 10, or 100 times the number of individuals in the actual data set. For experiments using the actual sampled light distributions (Figure S4), each individual was assigned its actual light level (these same light levels were duplicated as needed in the experiments with inflated sample sizes). In contrast, for experiments with a uniform light distribution, each individual was assigned a randomly generated light value from a uniform distribution ranging from zero to one (full sunlight).
